# Outcomes and use of healthcare resources after an intervention for chronic limb-threatening ischaemia

**DOI:** 10.1093/bjsopen/zrad112

**Published:** 2023-11-03

**Authors:** Athanasios Saratzis, Liam Musto, Santosh Kumar, Jingyi Wang, Louis Bojko, Joseph Lillington, Patrick Anyadi, Hany Zayed

**Affiliations:** Department of Cardiovascular Sciences, University of Leicester, Leicester, UK; Department of Cardiovascular Sciences, University of Leicester, Leicester, UK; National Health Service Health Economics Unit, NHS Midlands and Lancashire Commissioning Support Unit, Stoke on Trent, UK; National Health Service Health Economics Unit, NHS Midlands and Lancashire Commissioning Support Unit, Stoke on Trent, UK; National Health Service Health Economics Unit, NHS Midlands and Lancashire Commissioning Support Unit, Stoke on Trent, UK; National Health Service Health Economics Unit, NHS Midlands and Lancashire Commissioning Support Unit, Stoke on Trent, UK; National Health Service Health Economics Unit, NHS Midlands and Lancashire Commissioning Support Unit, Stoke on Trent, UK; School of Cardiovascular Sciences, King’s College, London, UK; Department of Vascular Surgery, Guy’s and St Thomas’ NHS Foundation Trust, London, UK

## Abstract

**Background:**

The fate of patients with chronic limb-threatening ischaemia undergoing revascularization or a primary amputation is unclear. The aim of this study was to assess the postoperative outcomes and post-procedural healthcare resource use/costs over 1 year after revascularization or a primary amputation for chronic limb-threatening ischaemia.

**Methods:**

The UK Kent Integrated Dataset, which links primary, community, and secondary care for 1.6 million people, was interrogated. All patients with a new diagnosis of chronic limb-threatening ischaemia undergoing revascularization or a major amputation between January 2016 and January 2019 (3 years) were identified. Postoperative events across all healthcare settings and post-procedure healthcare resource use were analysed over 1 year (until the end of 2019).

**Results:**

Overall, 4252 patients with a new diagnosis of chronic limb-threatening ischaemia were identified (65 per cent were male and the mean age was 73 years) between January 2016 and January 2019, of whom 579 (14 per cent) underwent an intervention (studied population); 296 (7 per cent) had an angioplasty, 75 (2 per cent) had bypass surgery, 141 (3 per cent) had a primary major lower limb amputation, 11 had a thrombo-embolectomy (0.3 per cent), and 56 had an endarterectomy (1.3 per cent). Readmissions (median of 2) were similar amongst different procedures within 1 year; bypass surgery was associated with more hospital appointments (median of 4 *versus* 2; *P* = 0.002). Patients undergoing a primary amputation had the highest number of cardiovascular events and 1-year mortality. In a linear regression model, index procedure type and Charlson co-morbidity index score were not predictors of appointments in primary/secondary care, community care visits, or readmissions after discharge. There were no statistically significant differences regarding post-procedural healthcare costs between procedures over 1 year.

**Conclusion:**

Revascularization is not associated with more hospital, primary/community care appointments or increased post-procedural healthcare costs over 1 year when compared with primary amputation, in people with chronic limb-threatening ischaemia.

## Teaser

The aim of this study was to use routinely available data, linking community, primary, and secondary care, to assess the fate of patients undergoing a primary amputation or revascularization for chronic limb-threatening ischaemia.

A revascularization strategy was not associated with higher costs or more postoperative healthcare appointments/events.

## Introduction

Peripheral artery disease (PAD) affects one-fifth of the population over the age of 55 years^1–3^. It is the most common cause of lower limb amputation and a leading cause of cardiovascular morbidity^4^. People with PAD who develop symptoms typically present either with intermittent claudication or chronic limb-threatening ischaemia (CLTI). More than half of those diagnosed with symptomatic PAD are expected to die, have an amputation, or have a major cardiovascular event within 5 years; those with CLTI are even more likely to suffer cardiovascular events or die^1,5–9^. People with CLTI are at a significant risk of limb loss or death without revascularization and therefore typically require urgent intervention. Such revascularization procedures might include endovascular treatment, open surgery, or a combination of both approaches (hybrid procedures). Given the co-morbidity burden of patients with CLTI and in cases where they are deemed unsuitable or unfit for revascularization, some patients might be offered a primary amputation. Previous research aimed to analyse the cost-effectiveness of revascularization in comparison with a primary amputation; however, many of these studies did not include the additional costs of postoperative care in the community and primary care settings, due to lack of availability of such data^10–14^.

The aim of this study was to define the natural history of people presenting with a new diagnosis of CLTI to secondary care and the overall impact on healthcare resource use, in terms of postoperative events, patient activity (across all healthcare environments), and resulting post-procedural costs.

## Methods

### Study design and data source

This analysis used routinely collected individual patient data from the Kent Integrated Dataset (KID); data are collected prospectively for consecutive patients presented to any public healthcare provider in the region covered. The KID represents a data partnership between Kent County Council Public Health and Kent & Medway Clinical Commissioning Groups. The data set is assembled by a data warehouse team hosted by an acute trust in Kent using data-sharing and processing arrangements with all local healthcare providers. The KID enables data analysis across primary and secondary care providers in Kent and Medway (population in 2020 of 1.6 million) integrating health and care de-identified data. This allows whole pathways to be analysed, including acute trusts, adult social care, general practitioner practices, mental health services, public health services, and community health services. Data on patients' activities and cost(s) are captured prospectively. The use of the KID helps provide an overall view of patients’ journeys before and after treatment in secondary care facilities. A data resource document is available outlining the linkages, contents, data validation, and quality checks^15^.

### Approvals

All data were de-identified (anonymized). No ethical approval was required to analyse this routinely collected fully anonymized information as per current National Health Service (NHS) Good Clinical Practice, Health Research Authority guidance, and the opinion of the NHS East Midlands (Leicester) NHS Research Ethics Committee. The research was funded in whole by Abbott Ltd (Maidenhead, UK)^16^. The funder was not involved in interpretation, data analysis, or reporting, and did not have access to the raw data.

### Study population (inclusion criteria)

Individuals greater than 18 years of age who presented with CLTI (Rutherford stages 4–6, that is the presence of rest pain and/or tissue loss in the lower limb) to secondary care and who underwent an intervention to address their CLTI during that presentation (first intervention for CLTI)^11,17^.

### Definitions

All definitions used in this study relating to clinical events and diagnoses were based on the Society for Vascular Surgery reporting guidelines for steno-occlusive PAD^11,17,18^. Clinical codes based on the ICD-10 codebook were used to identify diagnoses and clinical events; two authors (A.S. and H.Z.—both vascular surgeons) reviewed all relevant code lists to identify all relevant codes relating to baseline characteristics and postoperative events of interest^19^. With regards to identifying individuals with CLTI, people with a new diagnosis of PAD/atherosclerosis of the limb (codes I70.23-, I70.24-, I70.33-, I70.34-, I70.43-, I70.44-, I70.53-, I70.54-, I70.63-, I70.64-, I70.73-, and I70.74-), with or without a lower limb ulcer/gangrene/diabetic foot infection (not of venous nature; code L97. 909), referred to secondary care from primary care for an intervention, were searched for and those who presented with claudication as the only coding/identification parameter were excluded. Hybrid revascularization procedures were coded as open procedures. No individuals underwent a major amputation immediately after revascularization in this series/data set.

The objectives were as follows: to identify individuals with a new diagnosis of CLTI captured in the KID, who presented to secondary care between January 2016 and January 2019 (3 years) and underwent any of the following procedures during that presentation (index procedure): major lower limb amputation (that is amputation above the level of the ankle joint), bypass surgery (lower limb arterial bypass), endarterectomy, angioplasty, with or without stenting or other endovascular adjunctive procedure of the aorta and/or iliac artery and/or femoral artery and/or popliteal artery and/or any tibial artery, and thrombo-embolectomy of any artery of the lower limb (or the aorta and/or iliac artery); to describe the baseline co-morbidities/demographics of these individuals; and to report inpatient and outpatient outcomes, including all types of healthcare appointments, after these procedures.

The outcomes of interest were as follows: demographic characteristics, socioeconomic deprivation scores, and associated co-morbid conditions, including established cardiovascular risk factors (smoking, diabetes, hypertension, use of statins or ezetimibe, use of antiplatelet agents or other antithrombotic therapy, previous history of stroke, and ischaemic heart disease); duration of inpatient stay (in days) and potential in-hospital mortality; number of primary care appointments after discharge (after the index procedure); number and duration (in days) of secondary care readmissions after discharge (after the index procedure); mortality and major cardiovascular events (non-fatal myocardial infarction and/or non-fatal stroke or transient ischaemic attack and/or non-fatal heart failure) after discharge (after the index procedure); major limb amputations in those who had undergone revascularization; and post-procedural costs of care based on the number of primary care appointments and secondary care admissions in the first year post-procedure (index/main procedure).

### Follow-up

All events recorded in the KID between the index procedure for 1 year and until the end of December 2019 (that is before the COVID-19 pandemic) were recorded—1 year outcomes were recorded for all patients included in the analysis.

### Statistical analysis

Categorical variables are presented as absolute numbers and percentages of the total numbers for each subgroup. Continuous variables are presented as mean(s.d.) or median (range) for non-normally distributed variables. Normality of distributions was assessed graphically using plots and based on the Kolmogorov–Smirnov test. Statistical comparisons for categorical variables were conducted using the chi-squared test and statistical comparisons for continuous variables were conducted using the *t* test. The Mann–Whitney *U* test was used for non-normally distributed variables. Normality was assessed based on skewness, kurtosis, and graphical representation of the data. *P* values of <0.050 were considered statistically significant. Multivariable logistic and linear regression was used to identify the independent association between the patients’ postoperative activities, index procedure, and Charlson co-morbidity index (CCI) score^20^; predictor variables in the model included the CCI score, type of procedure, diabetes, dementia, presence of chronic kidney disease, and those that differed between groups and were thought to be of major clinical significance (in addition to all the parameters included in the CCI score, for example age/sex, etc.). Statistical analyses were performed using R version 4.2^21^. In particular, the ‘stats (version 3.6.2)’ and ‘dplyr (version 1.0.9)’ packages in R were used. An independent qualified statistician oversaw all statistical analyses based on a pre-specified plan.

### Calculation of care costs after the index procedure

All episodes within the KID include the costs of the episodes, which were used to calculate the sum of costs of the primary care, readmissions, outpatient appointments, and community appointments (including social care) within 3 months or 1 year after the day of the index procedure. Further, in this paper, these costs are referred to as post-procedural costs. If one patient had all costs under the certain type of care as blank (=NULL), it was treated as blank rather than ‘0’, but, if the patient had other costs with values under the certain type of care, it was added as ‘0’ in the sum. All episodes, regardless of indication, including those outside of vascular surgery, in the follow-up interval were counted towards the cost totals. The costs for each type of episode within the KID are derived from a variety of sources, depending on the episode type; for example the cost of primary care interactions is taken from the Personal Social Services Research Unit ‘Unit costs’ (which include costs per salary scale, consultation length, location, and overheads)^15,22^.

## Results

### Baseline characteristics

A total of 4252 patients with a new diagnosis of CLTI were identified in the KID (2762 (65 per cent) patients were male and the mean(s.d.) age was 73(11) years). During the study interval, the following index interventions/procedures took place amongst these patients: 296 patients (6.9 per cent) underwent an angioplasty, 75 patients (1.7 per cent) underwent bypass surgery, and 141 patients (3.3 per cent) underwent a major lower limb amputation as their index procedure (that is the first procedure to address CLTI upon presentation to secondary care). *Table 1* shows the baseline characteristics (demographics and co-morbidities) of patients undergoing an intervention who were included in the analysis based on their index procedures. Of interest, 156 (3.6 per cent) patients were current smokers, 152 (3.5 per cent) patients had diabetes (with or without chronic complications), and 77 (1.8 per cent) patients had chronic pulmonary disease.

**Table 1:** Baseline characteristics, including demographics and key co-morbidities

	Angioplasty	Endarterectomy	Bypass surgery	Major lower limb amputation	Thrombo- embolectomy	*P*
*n*	296	56	75	141	11	–
Age (years), mean(s.d.), median (range)	70(11), 71 (40–96)	71(8), 71 (52–87)	68(11), 68 (45–92)	71(13), 77 (32–93)	68(17), 73 (39–89)	0.001
Charlson co-morbidity index score, mean(s.d.), median (range)	3.5(2.4), 3 (0–13)	3.1(1.6), 3 (0–9)	2.9(2), 3 (0–10)	4.4(2.5), 4 (0–13)	3.5(2), 4 (0–6)	0.001
Patient sex (female/male)	93 (31.4)/203 (68.6)	18 (32)/38 (68)	18 (24)/57 (76)	43 (30.5)/98 (69.5)	3 (27)/8 (73)	0.359
Smoking status (ex-smoker/non-smoker/current smoker)	29 (9.8)/193 (65.2)/74 (25.0)	8 (14)/33 (59)/15 (27)	15 (2)/37 (49)/23 (31)	28 (19.9)/70 (49.7)/43 (0.7)	–/2 (18)/1 (9)	0.037
Co-morbidities						
Hypertension	109 (36.8)	20 (36)	27 (36.0)	31 (22.0)	1 (9)	0.465
Diabetes with chronic complications	56 (18.9)	5 (9)	10 (13)	42 (29.8)	1 (9)	0.036
Diabetes without chronic complications	13 (4.4)	2 (4)	1 (1)	24 (17.0)	1 (9)	0.239
Chronic pulmonary disease	44 (14.9)	5 (9)	5 (7)	22 (15.6)	1 (9)	0.020
Cerebrovascular disease	25 (8.5)	2 (4)	4 (5)	13 (9.2)	1 (9)	0.001
Congestive heart failure	23 (7.8)	2 (4)	3 (4.0)	15 (10.6)	–	0.381
Malignancy	11 (3.7)	–	4 (5)	3 (2.1)	–	0.283
Dementia	3 (1.0)	–	–	2 (1.4)	1 (9)	0.322
Myocardial infarction	5 (1.7)	2 (4)	5 (7)	13 (9.2)	–	0.320
Peripheral vascular disease	30 (10.1)	5 (0)	6 (8)	29 (20.6)	3 (27)	0.001
Peptic ulcer disease	3 (1.1)	–	1 (1)	2 (1.4)	1 (9)	0.001
Previous angioplasty	12 (4.1)	17 (30)	8 (11)	21 (14.9)	–	0.322
Rheumatic disease	5 (1.7)	–	1 (1)	5 (3.6)	1 (9)	0.392
Chronic kidney disease	17 (5.7)	23 (41)	19 (25)	41 (29.1)	2 (18)	0.004

Values are *n* (%) unless otherwise indicated.

### Inpatient outcomes

A total of 206 (5.2 per cent) patients died during their inpatient stay. The median inpatient stay after each type of procedure within 1 year (described in *Table 2*) was as follows: major lower limb amputation, 13 (range 0–159) days; bypass surgery, 15 (range 0–103) days; endarterectomy, 6 (range 0–117) days; angioplasty, 7 (range 0–225) days; and thrombo-embolectomy, 11 (range 2–119) days.

**Table 2:** Postoperative inpatient stay and subsequent patient appointments after each index procedure

	Angioplasty	Endarterectomy	Bypass surgery	Major lower limb amputation	Thrombo- embolectomy	*P*
*n*	296	56	75	141	11	–
Duration of postoperative stay (days)	1 (0–111)	4 (1–128)	8 (2–109)	30 (0–166)	7 (0–58)	0.631
Readmissions to hospital within 1 year	2 (1–18)	2 (1–17)	2 (1–9)	2 (1–10)	1 (1–14)	0.833
Duration of stay during readmission within 1 year (days)	7 (0–255)	6 (0–117)	15 (0–103)	13 (0–159)	11 (2–119)	0.730
Community care appointments within 1 year	20 (1–678)	18 (2–239)	16 (1–259)	27 (1–613)	15 (3–18)	0.428
Outpatient hospital appointments within 1 year	4 (1–44)	5 (1–21)	4 (1–16)	4 (1–41)	3 (1–24)	0.003

Values are median (range) unless otherwise indicated. A median of 2 appointments post-bypass surgery were graft surveillance appointments.

### Outpatient outcomes

The numbers of community and primary care appointments after each index procedure within 1 year are described in *Table 2*. Patients undergoing a major lower limb amputation had the highest number of community care appointments within 1 year (median of 27 appointments), followed by patients undergoing an angioplasty (median of 20 appointments), but differences were not statistically significant (*P* = 0.428). Patients who had an endarterectomy had the most outpatient hospital appointments (median of 5 appointments) within 1 year *versus* 4 appointments for angioplasty and major lower limb amputation (*P* = 0.002). A total of 204 patients required at least one readmission to hospital within 3 months and a total of 322 patients required at least one readmission to hospital within 1 year. *Table 2* summarizes the number of readmissions and postoperative appointments per index procedure type, as well as the duration of stay during readmission. Apart from the number of outpatient hospital appointments after discharge, there were no statistically significant differences between types of index procedures for number of readmissions, duration of stay during readmission, or community care appointments. Major cardiovascular events and lower limb amputations after each index procedure within 1 year are summarized in *Table 3*. Patients undergoing a major lower limb amputation had the highest number of cardiovascular events within 1 year (mean of 3.9 events) and patients undergoing an endarterectomy had the highest number of lower limb amputations within 1 year (*Table 3*). Overall, 51 amputations (major) took place in those who had revascularization, over 1 year (11.6 per cent).

**Table 3:** Major cardiovascular events, lower limb amputations, and all-cause mortality after each index procedure

	Angioplasty	Endarterectomy	Bypass surgery	Major lower limb amputation	Thrombo- embolectomy	*P*
*n*	296	56	75	141	11	–
Major cardiovascular events within 1 year, median (range)	1 (1–5)	2 (1–3)	1 (1–1)	3 (1–3)	–	0.611
Any lower limb amputation within 1 year, median (range)	1 (1–2)	1 (1–1)	1 (1–1)	1 (1–2)	1 (1–1)	0.400
All-cause mortality within 1 year	50 (16.9)	10 (18)	12 (16)	45 (31.9)	5 (46)	0.001

Values are *n* (%) unless otherwise indicated.

In a linear regression model, the type of index procedure and CCI score were not predictors of patients’ activities (the total number of outpatient appointments in primary care, community care, or readmissions) (*Table 4*).

**Table 4:** Linear regression model to assess the potential impact of each type of intervention on post-intervention number of primary, secondary, and community care appointments during follow-up

	Linear regression model (coefficients)	*P*
Endarterectomy	−3.511	0.685
Bypass surgery	−0.108	0.988
Major lower limb amputation	3.202	0.607
Thrombo-embolectomy	−20.083	0.270
Charlson co-morbidity index score	4.703	0.001

### Costs of postoperative care

Patients undergoing a major lower limb amputation incurred the highest costs for postoperative community care within 1 year and patients undergoing a thrombo-embolectomy incurred the highest costs for postoperative primary care within 1 year (*Table 5*). All relevant post-procedural costs are summarized per procedure type in *Table 5*.

**Table 5:** Healthcare costs (in euros) incurred per procedure type

	Angioplasty	Endarterectomy	Bypass surgery	Major lower limb amputation	Thrombo- embolectomy	*P*
*n*	296	56	75	141	11	–
Costs secondary to hospital readmission within 3 months	4041 (0–22 834)	3485 (0–17 476)	5007 (349–17 075)	4217.5 (370–17 181)	6465.5 (577–14 403)	0.006
Costs secondary to hospital readmission within 1 year	5869 (0–58 405)	5076 (394–22 945)	6331 (349–36 040)	5773 (338–34 365)	10 799 (1075–17 483)	0.338
Community care costs within 3 months	615 (32–6393)	648 (32–4018)	636 (32–3982)	579 (32–4938)	349 (128–734)	0.004
Community care costs within 1 year	925 (32–27 706)	933 (80–9701)	848 (37–10 692)	1204 (32–25 842)	724 (141–874)	0.710
Primary care costs within 3 months	154 (18–1435)	143 (28–899)	228 (8–868)	155 (18–775)	434 (213–465)	0.700
Primary care costs within 1 year	245.5 (17.8–5129)	292.9 (17.8–3308)	303 (17.8–2139)	164 (17.8–2139)	1126 (97–1395)	0.813

Values are median (range) unless otherwise indicated. The date of conversion from British pounds to euros was 28 July 2023.

## Discussion

This study used routinely available data across the whole healthcare spectrum in one broad UK region to report outcomes, healthcare activity, and overall post-procedural-related costs to the healthcare system of people undergoing an intervention for CLTI. Information that links all of their community, primary, and secondary care interactions was used. Previous data sets for CLTI have not linked community, primary, and secondary care activities alongside procedural parameters and patient characteristics, which makes the findings of this study unique^11,23^. The findings suggest that patients undergoing a major lower limb amputation incur high costs for postoperative care within 1 year, especially at the community level. Post-procedural-related healthcare costs after an index amputation were similar to those of revascularization procedures. In fact, patients undergoing primary revascularization (for example angioplasty, bypass surgery, or endarterectomy) to salvage their limb incurred less post-procedural costs compared with those undergoing a primary amputation (this did not reach significance). Most importantly, those undergoing revascularization did not require significantly more hospital or primary/community care appointments over 1 year. Furthermore, even when adjusted for baseline CCI score, treating patients with an intent to salvage their limb (that is offer of revascularization rather than a primary amputation) did not lead to more deaths or more post-procedural appointment costs.

Consecutive patients with a new diagnosis of CLTI were identified in the KID (ischaemic rest pain or tissue loss in the background of PAD) based on the search strategy during the 3 year study interval. As expected, not every patient with a new CLTI diagnosis underwent an intervention to address their CLTI in secondary care. Of those with a CLTI diagnosis in the aforementioned interval, 6.7 per cent underwent an angioplasty, 1.9 per cent underwent bypass surgery, and 3.5 per cent underwent a major lower limb amputation as their index procedure. This study focused exclusively on CLTI patients who underwent an intervention. Outcomes for patients who did not undergo an intervention were beyond the scope of this work and were not explored. The large proportion of new CLTI patients not undergoing an intervention during their index admission may be reflective of regional practice and the burden of patient frailty and morbidity in those diagnosed with CLTI. As demonstrated in previous research, revascularization is often delayed or deferred from the index admission in the NHS and has a varying impact on patient outcomes^24^. A population-based study of UK general practice data demonstrated that CLTI patients within the NHS often present late to secondary care, often after several missed opportunities in primary and community care to diagnose their severe PAD early and allow timely intervention^25^. The study interval of the present study occurred at the same time as the publication of the Get It Right First Time (GIRFT) report (2018) and before the implementation of national targets to improve these aspects of patient care^26–28^ as reflected in the Commissioning for Quality and Innovation scheme.

Treatment for CLTI in the NHS is offered in specialized vascular centres and these patients would have been referred to the main vascular site (hub) for their corresponding area. Even so, 3.5 per cent of all patients with a new CLTI diagnosis in the KID over the 3-year window of this study were treated with a primary amputation rather than some form of revascularization (angioplasty or surgery). This might be due to patients’ preferences, co-morbidities, or the un-salvageable nature of the limb in some cases, possibly as a result of the non-optimal patient referral pathway. In any case, this is a high proportion, especially for a developed country, with considerable investment in healthcare.

As expected, the CCI score of patients undergoing a primary amputation was higher than that of patients treated with the intention of limb salvage (that is revascularization of some nature); however, the CCI score itself was not associated with more re-interventions or healthcare appointments. The co-morbid conditions of the patients who were offered an intervention were characteristic of a cohort with established CLTI, with high rates of smokers and prevalence of cardiovascular conditions, regardless of the type of procedure these patients were offered. This confirms that the current analysis represents a pragmatic ‘real-world’ snapshot of patients with severe PAD undergoing an intervention in a hospital setting. As discussed, CCI score was not predictive of post-procedure primary/secondary care activities/appointments, regardless of index procedure type.

An important message of this study is the lack of glaring differences, in terms of the post-procedural healthcare costs and appointments, when patients with CLTI are offered an intervention to salvage their limb and not a primary amputation. Recent studies have revealed comparable results, specifically in terms of total inpatient and human resource expenses^13^. Simulated studies predicting costs for a 10-year interval estimated fairly equivalent costs across all strategies (bypass, endovascular, and amputation), leaning towards surgical bypass as the most cost-effective option in a setting of more costly endovascular procedural fees and citing primary amputation as the least cost-effective of the three key strategies^14^.

Proponents of treating patients with advanced PAD/CLTI with a primary amputation cite fewer re-interventions and postoperative appointments as a cause for adopting such an approach^23,29^. The current data do not support the assumption that a primary amputation is less costly and burdensome than revascularization. They demonstrate that a major lower limb amputation is associated with a considerable burden after surgery, in terms of readmissions, appointments in primary/community, and secondary care readmissions. This should be taken into consideration when deciding what treatment options should be offered to patients with CLTI and when.

The analysis is based on routinely collected observational (non-randomized) data, risking the introduction of selection bias. This was not a prospective study, which might have an impact on data accuracy. At the same time, the KID is validated and includes information across all healthcare settings, which is unique for data sets of this nature globally. The KID does not capture the whole of the NHS or include international data, which has an impact on the generalizability of these findings. Despite this, the KID captures a considerable geographical population in the south of England (1.6 million people), with diverse backgrounds, treated across a variety of healthcare settings. Anatomy and in-depth procedural details are not available for this group of patients, as this was not captured in the data set and could not be interrogated retrospectively. Due to the absence of distinct coding for hybrid procedures in the data set, it was not possible to determine the number of hybrid procedures conducted in the data set. Hybrid procedures were coded as open surgery, as this would be the primary procedure indicated within the data set and may artificially inflate the open surgical numbers reported. Although it was possible to determine staff grade (for example ‘doctor’ or ‘other’), it was not possible to confirm if the diagnosis was confirmed by a vascular surgeon in secondary care. Further economic analysis, including capturing the index procedure costs, as well as costs of cardiovascular events occurring during the follow-up interval, is recommended in order to provide a complete picture of the overall economic impact of each type of intervention.

Finally, information for those treated without an intervention was not captured and was outside the scope of this study, and the overall case-capture proportion using the KID as a tool cannot be assessed. Within its limitations, this study shows that a strategy of adopting revascularization rather than primary amputation is not associated with more post-procedural hospital or primary care/community care appointments or increased post-procedural-related costs.

## Data Availability

Raw data are not available without ethical approval due to restrictions by the Kent Integrated Dataset (KID). Fully anonymized data are available after request to the corresponding author.

## References

[1] Morley RL, Sharma A, Horsch AD, Hinchliffe RJ. Peripheral artery disease. BMJ 2018;360:j5842 29419394 10.1136/bmj.j5842

[2] National Institute for Health and Care Excellence . *Peripheral Arterial Disease: Diagnosis and Management, Guidance, Overview*. 2020. https://www.nice.org.uk/guidance/cg147 (accessed 20 March 2023)

[3] Cea-Soriano L, Fowkes FGR, Johansson S, Allum AM, García Rodriguez LA. Time trends in peripheral artery disease incidence, prevalence and secondary preventive therapy: a cohort study in The Health Improvement Network in the UK. BMJ Open 2018;8:e018184 10.1136/bmjopen-2017-018184 PMC5780686 29358428

[4] Meffen A, Pepper CJ, Sayers RD, Gray LJ. Epidemiology of major lower limb amputation using routinely collected electronic health data in the UK: a systematic review protocol. BMJ Open 2020;10:e037053 10.1136/bmjopen-2020-037053 PMC7295407 32532778

[5] Parvar SL, Fitridge R, Dawson J, Nicholls SJ. Medical and lifestyle management of peripheral arterial disease. J Vasc Surg 2018;68:1595–1606 30360849 10.1016/j.jvs.2018.07.027

[6] Dhaliwal G, Mukherjee D. Peripheral arterial disease: epidemiology, natural history, diagnosis and treatment. Int J Angiol 2007;16:36–44 22477268 10.1055/s-0031-1278244 PMC2733014

[7] Dua A, Lee CJ. Epidemiology of peripheral arterial disease and critical limb ischemia. Tech Vasc Interv Radiol 2016;19:91–95 27423989 10.1053/j.tvir.2016.04.001

[8] Nehler MR, Duval S, Diao L, Annex BH, Hiatt WR, Rogers K et al Epidemiology of peripheral arterial disease and critical limb ischemia in an insured national population. J Vasc Surg 2014;60:686–695.e2 24820900 10.1016/j.jvs.2014.03.290

[9] Saratzis A, Jaspers NEM, Gwilym B, Thomas O, Tsui A, Lefroy R et al Observational study of the medical management of patients with peripheral artery disease. Br J Surg 2019;106:1168–1177 31259387 10.1002/bjs.11214

[10] Farber A, Menard MT, Conte MS, Kaufman JA, Powell RJ, Choudhry NK et al Surgery or endovascular therapy for chronic limb-threatening ischemia. N Engl J Med 2022;387:2305–2316 36342173 10.1056/NEJMoa2207899

[11] Conte MS, Bradbury AW, Kolh P, White JV, Dick F, Fitridge R et al Global vascular guidelines on the management of chronic limb-threatening ischemia. J Vasc Surg 2019;69(Suppl):3S–125S.e40 31159978 10.1016/j.jvs.2019.02.016 PMC8365864

[12] Dillingham TR, Pezzin LE, Shore AD. Reamputation, mortality, and health care costs among persons with dysvascular lower-limb amputations. Arch Phys Med Rehabil 2005;86:480–486 15759232 10.1016/j.apmr.2004.06.072

[13] Popplewell MA, Andronis L, Davies HOB, Meecham L, Kelly L, Bate G et al Procedural and 12-month in-hospital costs of primary infra-popliteal bypass surgery, infrapopliteal best endovascular treatment, and major lower limb amputation for chronic limb threatening ischemia. J Vasc Surg 2022;75:195–204 34481898 10.1016/j.jvs.2021.07.232

[14] Barshes NR, Chambers JD, Cohen J, Belkin M. Cost-effectiveness in the contemporary management of critical limb ischemia with tissue loss. J Vasc Surg 2012;56:1015–1024.e1 22854267 10.1016/j.jvs.2012.02.069

[15] Lewer D, Bourne T, George A, Abi-Aad G, Taylor C, George J. Data resource: the Kent Integrated Dataset (KID). Int J Popul Data Sci 2018;3:427 32935003 10.23889/ijpds.v3i1.427 PMC7299463

[16] Abbott . *Contacts*. https://www.abbott.co.uk/contact.html (accessed 27 March 2023)

[17] Wanhainen A, Verzini F, Van Herzeele I, Allaire E, Bown M, Cohnert T et al Editor’s Choice – European Society for Vascular Surgery (ESVS) 2019 clinical practice guidelines on the management of abdominal aorto-iliac artery aneurysms. Eur J Vasc Endovasc Surg 2019;57:8–93 30528142 10.1016/j.ejvs.2018.09.020

[18] Stoner MC, Calligaro KD, Chaer RA, Dietzek AM, Farber A, Guzman RJ et al Reporting standards of the Society for Vascular Surgery for endovascular treatment of chronic lower extremity peripheral artery disease: executive summary. J Vasc Surg 2016;64:227–228 27345507 10.1016/j.jvs.2016.03.432

[19] ICD . *International Statistical Classification of Diseases and Related Health Problems 10th Revision, ICD-10 Version:2019*. 2019. https://icd.who.int/browse10/2019/en#/ (accessed 20 March 2023)

[20] Charlson ME, Carrozzino D, Guidi J, Patierno C. Charlson comorbidity index: a critical review of clinimetric properties. Psychother Psychosom 2022;91:8–35 34991091 10.1159/000521288

[21] R Core Team . *R: A Language and Environment for Statistical Computing*. 2022. https://www.R-project.org/

[22] Personal Social Services Research Unit . *Unit Costs of Health and Social Care 2016*. https://www.pssru.ac.uk/project-pages/unit-costs/unit-costs-2016/ (accessed 20 March 2023)

[23] Sibona A, Bianchi C, Leong B, Caputo B, Kohne C, Murga A et al A single center’s 15-year experience with palliative limb care for chronic limb threatening ischemia in frail patients. J Vasc Surg 2022;75:1014–1020.e1 34627958 10.1016/j.jvs.2021.09.032

[24] Li Q, Birmpili P, Johal AS, Waton S, Pherwani AD, Boyle JR et al Delays to revascularization for patients with chronic limb-threatening ischaemia. Br J Surg 2022;109:717–726 35543274 10.1093/bjs/znac109 PMC10364726

[25] Nickinson ATO, Coles B, Zaccardi F, Gray LJ, Payne T, Bown MJ et al Missed opportunities for timely recognition of chronic limb threatening ischaemia in patients undergoing a major amputation: a population based cohort study using the UK’s clinical practice research datalink. Eur J Vasc Endovasc Surg 2020;60:703–710 32718828 10.1016/j.ejvs.2020.05.010

[26] The Vascular Society for Great Britain and Ireland . *A Best Practice Clinical Care Pathway for Peripheral Arterial Disease*. 2019. https://www.vascularsociety.org.uk/_userfiles/pages/files/Document%20Library/VS%202018%20Final.pdf (accessed 27 March 2023)

[27] The Vascular Society for Great Britain and Ireland . *A Best Practice Clinical Care Pathway for Peripheral Arterial Disease*. 2022. 10.54522/jvsgbi.2022.17 (accessed 27 March 2023)

[28] Horrocks M . *Vascular Surgery, GIRFT Programme National Specialty Report*. 2018

[29] Tang L, Paravastu SCV, Thomas SD, Tan E, Farmer E, Varcoe RL. Cost analysis of initial treatment with endovascular revascularization, open surgery, or primary major amputation in patients with peripheral artery disease. J Endovasc Ther 2018;25:504–511 29756521 10.1177/1526602818774786

